# Supplementary material for: Getting More Out of Biomedical Documents with GATE's Full Lifecycle Open Source Text Analytics
Source: PLoS Comput Biol. 2013 Feb 7;9(2):e1002854. doi: 10.1371/journal.pcbi.1002854 (PMC3567135; doi:10.1371/journal.pcbi.1002854)
Supplement: Dataset S1 — GATE software. Dataset S1 bundled with this paper contains a distribution of GATE (or see http://gate.ac.uk/download/). (TGZ) [file pcbi.1002854.s001.tgz › plos-gate/plugins/Gazetteer_LKB/doc/comparison.html]

The Large KB gazetteer - GATE Gazetteer Feature Comparison


---

Last Published: 2009-10-17

---

##### Large KB Gazetteer

- Overview
- Usage
- Q & A
- GATE Compatibility
- **GATE Gazetteer Feature Comparison**
- Semantic Enrichment PR
- Samples

##### Developer Documentation

- Dictionary Lifecycle

There is already a variety of gazetteer implementations in GATE. This page will contain details feature and / or performance comparison will all that implementations. This is a work in progress. Contact the team if you need immediate information on that topic.

---

©
2009
Ontotext AD

---
